# Phytochemical and pharmacological evaluation of ethanolic extract of *Lepisanthes rubiginosa* L. leaves

**DOI:** 10.1186/s12906-017-2010-y

**Published:** 2017-11-22

**Authors:** Md. Mahedi Hasan, Amir Hossain, Abdullah Shamim, Md. Mustafizur Rahman

**Affiliations:** 1 Pharmacy Discipline, Life Science School, Khulna University, Khulna, 9208 Bangladesh; 2 Department of Pharmacy, Dhaka International University, Satarkul, Badda, Dhaka, Bangladesh

**Keywords:** *Lepisanthes rubiginosa*, Antioxidants, Analgesic, Antihyperglycemic, Antidiarrheal, Neuropharmacological, Mice

## Abstract

**Background:**

The current study was conducted to evaluate the antioxidant, analgesic, antihyperglycemic, neuropharmacological and antidiarrheal activities of ethanolic extract of *Lepisanthes rubiginosa* L. leaves in different experimental models.

**Methods:**

Quantitative and qualitative analysis were done by TLC (thin layer chromatography) and DPPH (1,1-diphenyl-2-picrylhydrazyl) free radical scavenging assay. Analgesic, antihyperglycemic and antidiarrheal activities were evaluated using acetic acid induced writhing in mice, oral glucose tolerance test and castor oil induced diarrhea, respectively. Neuropharmacological activity was investigated in mice using both Open Field and Hole Board methods.

**Results:**

TLC analysis indicated the presence of antioxidant compounds in the extract we used. The extract showed IC_50_ value was 31.62 μg/mL whereas the standard ascorbic acid showed 12.02 μg/mL. In acetic acid induced writhing assay, the extract showed 46.07% and 58.43% writhing inhibition at the doses of 250 mg/kg and 500 mg/kg body weight, respectively whereas standard diclofenac-Na (25 mg/kg) showed 86.52% writhing inhibition. The plant extract showed significant (*p* < 0.05) antihyperglycemic activity on mice as compared to control groups. In neuropharmacological activity assay the experimental animal showed a noticeable decrease in locomotion by showing a decrease in number of square crossed and head dipping at both doses (250 mg/kg & 500 mg/kg). In antidiarrheal activity test, the plant extract at the doses of 250 mg/kg and 500 mg/kg showed percent inhibition of defecation 57.89 and 77.19 respectively, whereas standard loperamide (3 mg/kg) showed percent inhibition of defecation 88.59.

**Conclusion:**

The results demonstrated that the extract has potential antioxidant, analgesic, antihyperglycemic, neuropharmacological and antidiarrheal activity.

**Electronic supplementary material:**

The online version of this article (10.1186/s12906-017-2010-y) contains supplementary material, which is available to authorized users.

## Background

Everyday researchers are searching for new drugs with better or improved therapeutic actions. Medicinal plants provide an excellent source of lead compound in discovering noble and new drugs with considerable less side or adverse effects [1]. Traditional medicines are very important since early ancient period because of their faithfulness in the use of various ailments and human sufferings. Various types of bioactive natural compounds are derived from medicinal plants and it serves as raw materials for new drug discovery [2]. Different types of phytochemicals such as alkaloids, saponins, carbohydrates, glycosides, flavonoids, gums, steroids, tannins, phenolic compounds, volatile oils etc. are synthesized from numerous types of medicinal plants that have a potential therapeutic and pharmacological activities and are used in different disorders [3]. In last few years, there has been great focus on the possible health benefits of natural substances with antioxidant, antimicrobial, analgesic, anticancer, anti-diabetic and others activity. This has resulted in an enormous increase of research on different medicinal plant to find lead compounds responsible for such pharmacological activity.

*L. rubiginosa* is evergreen shrub and height usually 2–3 m, sometimes to 7 m. The plants have been traditionally used for various purposes such as antipruritic, headache, fever, decoction, antitussive, tonics and food preservatives. Stems are used for inducing sleep. Saburi et al. carried out chemical investigation of the methanolic fraction of *L. rubiginosa* bark which led to the isolation and characterization of a new tetrasaccharide derivative of farnesol named rubiginoside along with known triterpenoid saponins [4]. In a study, it is seen that an aqueous fruit extract of *L. rubiginosa* at doses of 20 mg/kg and 100 mg/kg significantly (*p* < 0.05) decrease locomotion and at dose of 100 mg/kg enhanced the thiopental-induced sleeping period. In another study, it was also disclosed the affinity to the dopaminergic receptors and inhibition of the apomorphine induced climbing behavior in experimental mice [5, 6]. In that consequence, it is assumed that the leaf extract should exert neuropharmacological activity, and hence, we carried out in-vivo experiments to evaluate neuropharmacological activity.

The drugs which are currently used as antidiabetics have serious side effects and deleterious contraindications [7]. Hence, researchers are paying attention to herbal medications having high therapeutic efficacy with minimal side effects. The antidiabetic agents from medicinal plants are very promising and traditionally acclaimed medicinal plants are being investigated for their antidiabetic potential [8, 9]. In other studies, the fruit extracts of some plants were evaluated for alpha-amylase, alpha-glucosidase, total phenolic and total flavonoid contents where the extract have shown significant anti-hyperglycemic and analgesic activities [10–12]. In this context, we hypothesize that the leaf extracts of this plant might possess compounds with anti-hyperglycemic and analgesic activities.

Alkaloids, flavonoids, phenolics, tannins, saponin, glycosides and steroids were identified in the preliminary phytochemical investigation of *Lepisanthes rubiginosa* leaf extract. Researchers have reported a number of bioactive compounds from different plants to have antihyperglycemic effect among which mostly phenolics, flavonoids etc. have a positive correlation as antidiabetic agents [13–15]. Our phytochemical screening identified the presence of flavonoids, phenolics in our leaf extract of Lepisanthes rubiginosa. And this prompted us to investigate the antihyperglycemic activity of the extract using in vivo model.

Among different disorders, diarrhea is one of the causes of morbidity and mortality specially in developing countries [16]. The world health organization (WHO) has established a diarrhea disease control program for the treatment and management of diarrhea that includes traditional medicine practices along with health education and prevention approaches (Syder medicine), which is mostly based on herbal products [17]. WHO has accepted traditional medicine as an alternative health care form. In a developing country like Bangladesh where a handsome amount of people including children are affected by diarrhea every year, a search for plants with antidiarrheal activity that could be used against diarrheal disease is of prime interest. However, amazingly no research has been carried out to evaluate the in-vivo antidiarrheal activity of the leaves extract of *L. rubiginosa.*

In a comparative study of the essential oil from flowers and fruits of *L. rubiginosa*, the flower essential oil showed anticancer activity against NCI-H187 (small cell lung cancer) and possessed antioxidant activity. But fruit essential oil did not show anticancer activity and possessed low antioxidant activity. However, both flower and fruit essential oils showed strong antimicrobial activity against *Tricophytonmentagrophytes* and moderate activity against *E. coli*, *S. aureus*, *Pseudomonas aeruginosa* and *Candida albicans* [6]. The findings of the above-mentioned studies formed the basis of our hypothesis that the leaf extract of the plant could have bioactivity beneficial for human health. And thus, we have been prompted to carry out in vivo evaluation of the leaf extract of the plant in animal models. And hence, the current study was designed to evaluate the antioxidant activity, neuropharmacological, analgesic, antihyperglycemic and antidiarrheal activities of ethanolic extract of *L. rubiginosa* leaves in different experimental models.

## Methods

### Collection of plant material

The leaves of *L. rubiginosa* were used as the raw material for the extraction and other investigation process. The leaves of *L. rubiginosa* were collected from the Sundarbans, Bangladesh in April, 2014. Any types of undesirable materials or plants or plant parts were separated from the collected plant parts. The plant identification was authenticated by Dr. HosneAra, Director, Bangladesh National Herbarium (BNH), Mirpur-1, Dhaka-1216. A voucher specimen (Accession number: DACB-44952) was deposited at the Herbarium.

### Drying and grinding

The collected plant parts were washed with water. After washing it was subjected to shed-drying for 1–2 weeks. When the plant parts were suitable for grinding, it was grinded into coarse powder by a grinder (Wuhu motor factory, China). Finally, the powder material was stored in a sealed container and kept in a dark, cool and dry place until farther processing.

### Cold extraction

About 250 g of finely powered plant materials was taken in a clean glass container and it was soaked with 800 mL of C_2_H_5_OH. Then the container was sealed and kept for a period of 15 days. During this time, it was subjected to occasional stirring and shaking. The mixture was then filtered by cotton. Finally, it was filtered by Whatman filter paper.

### Experimental animals

About 4–5 weeks aged Swiss-albino mice of both sexes (Around 50 mice), average weight of 20–35 g was collected from central animal house of the Department of Pharmacy, Jahangirnagar University, Savar, Dhaka-1342, and were used for the present study. The animals were randomly selected and divided into normal and experimental groups. The research was carried out according to the rules governing the use of laboratory animals and the experimental protocol was approved by the Animal Ethics Committee, Khulna University. After one week for their adaptation, all the experimental processes would be conducted and it would be performed in an isolated and noiseless environmental condition. All sections of this report adhere to the ARRIVE Guidelines for reporting animal research. A completed ARRIVE guidelines checklist is included with the submission as Additional file 1.

### Chemicals and reagents

Acetone, n-hexane, chloroform, methanol, ethanol, ascorbic acid, quercetin, sodium nitrous, aluminum chloride, sodium hydroxide, sodium carbonate, gallic acid, acetic acid, 2, 2-diphenyl-1-picrylhydrazyl (DPPH), and folin-ciocalteu’s reagent were obtained from Sigma Chemical Co. Ltd., St. Louis, MO, USA. Castor oil was supplied by LobaChemie Pvt. Ltd., India.

### Reference drugs

Loperamide and diclofenac-Nawere purchased from Square Pharmaceuticals Ltd., Bangladesh. Diazepam and Glibenclamide were purchased from Incepta Pharmaceuticals Ltd., Bangladesh.

### Evaluation of antioxidant activity

Ethanol leaves extract Of*l. rubiginosa* was evaluated for antioxidant activity by both qualitative and quantitative method. Thin-layer chromatographic (TLC) technique was used for qualitative analysis and DPPH-scavenging technique was used for quantitative analysis [18].

### Qualitative analysis

Thin-layer chromatography (TLC) is a chromatographic technique that is useful for separating wide range of organic compounds. Maximum diluted solutions of extract were spotted on TLC plates and then the plates were developed with different type of solvent systems like polar, medium polar and non-polar to separate polar and non-polar components from the plant materials. When the chromatogram of the extract was developed, it forms pale yellow to the yellow color that indicate the presence of antioxidant components [19].

### Quantitative analysis

DPPH, having an odd number of electrons is a stable free radical. Different concentrations (1–512 μg/mL) of the extract were taken in different test tubes and 3 ml of a 0.004% *w*/*v* solution of DPPH were added to it. After 30 min, absorbance was taken at 517 nm, and IC_50_ (Inhibitory concentration 50%) was determined. IC_50_ value indicates the concentration of sample required to scavenge 50% of the DPPH free radicals [19]. Here, ascorbic acid was used as a standard compound. Percent inhibition was calculated using the following formula:

*Percent inhibition* = (1 – *A*_1_/*A*_0_) × 100 % ,

where A_0_ is the absorbance of blank and A_1_ is the absorbance of sample or standard.

### Determination of total phenolic content

The total phenolic content of the ethanolic leaves extract of *L. rubiginosa* was determined by using Folin-Ciocalteu’s reagent [20–23]. Gallic acid (12.5 mg) was dissolved in methanol to make final volume up to 25 ml. Different concentrations (500–15.62 mg/L) of standard gallic acid solution was made by serial dilution method. 1 ml solution of every concentration was taken into different volumetric flask and 9 ml of distilled water along with 1 ml of FC reagent (10% *v*/v) was added to it. After 5 min, 10 ml solution of 7% sodium carbonate (Na_2_CO_3_) was added every volumetric flasks and volume was adjusted to make final volume up to 25 ml. After 30 min, absorbance was observed at 750 nm against the blank solution. Total phenolic content of the extract was evaluated against gallic acid standard calibration curve and expressed as mg gallic acid equivalent, GAE/100 g of dried plant extract.

### Determination of total flavonoid content

Ethanolic extract of *L. rubiginosa* leaves was evaluated for total phenolic content by using quercetin standard calibration curve [23–25]. Plant extract (10 mg) was dissolved with 10 ml distilled water in order to get final concentration 1 mg/1 ml. 0.1, 0.08, 0.06, 0.04 and 0.02 mg quercetin was mixed in 1 ml of distilled water to make the final concentrations 100, 80, 60, 40 and 20 μg/ml. 1 ml quercetin solution of every concentration (100-20 μg/ml) was taken into different volumetric flask and 4 ml of distilled water was added to it. Then 0.3 ml 5% *v*/v sodium nitrous solution was added. After 5 min, 2 ml 1 M sodium hydroxide (NaOH) was added and final volume was made up to 10 ml. Finally, UV absorbance was determined at 510 nm against blank solution. Total flavonoid content of the extract was also measured using quercetin standard calibration curve and expressed as mg quercetin equivalent, QE/100 g of dried plant extract.

### Determination of total tannin content

Ethanolic extract of *L. rubiginosa* leaves was evaluated for total phenolic content by using Folin-Ciocalteu’s reagent [26, 27]. Plant extract (10 mg) was dissolved in 10 ml distilled water to make final concentration of 1 mg/1 ml. Different aliquots of gallic acid (0.5, 0.4, 0.3, 0.2 and 0.1 mg) was dissolved in 1 ml of distilled water to make the final concentrations 100, 80, 60, 40 and 20 μg/ml. After preparing 3.5% *w*/*v* sodium carbonate solution, 0.1 ml gallic acid solution of every concentration (100-20 mg/l) was taken into different volumetric flask. Then 7.5 ml of distilled water and 0.5 ml FC reagent was added to it. After 5 min, 1 ml 3.5% sodium carbonate solution and 10 ml distilled water was added to that flask. After 30 min, UV absorbance was determined at 725 nm. Total tannin content of the extract of *L. rubiginosa* was measured using gallic acid standard calibration curve and stated as mg gallic acid equivalent, GAE/100 g of dried plant extract.

### Evaluation of analgesic activity

To investigate analgesic activity, the experimental animals were divided into four groups: control, standard and two test samples consisting of five mice of each group. The control groups received only vehicle (1% tween-80 water) whereas test groups received extract doses of 250 mg/kg and 500 mg/kg body weight of mice. Diclofenac-Na (25 mg/kg) was used as a standard drug [28, 29]. Control, standard, extract doses of 250 mg/kg and 500 mg/kg were given orally with the help of a feeding needle. After 30 min, 0.7% *v*/v acetic acid solution was administered intraperitoneally by all groups. After an interval of 5 min, number of writhing was counted for 15 min [30]. The mice do not always give full writhing. Sometimes they begin to produce writhing but they are not complete it. This incomplete writhing was then count as half writhing, so two half writhing was equal to one full writhing.

### Evaluation of antihyperglycemic activity

In antihyperglycemic activity test, the experimental animals were divided into four groups: control, standard and two test samples consisting of five mice of each group. The control groups received only vehicle (1% tween-80 water) whereas test groups received extract doses of 250 mg/kg and 500 mg/kg body weight of mice. Glibenclamide (5 mg/kg) was used as a standard drug. In a fasting state (having no food for at least 10 h but not more than 16 h), the experimental animals were tested. In a fasting state (having no food for at least 10 h but not more than 16 h), the experimental animals were tested. After selection and weighing of mice, fasting blood glucose level for control, standard and two test groups were measured. Then glucose solution was administered orallyand blood glucose levels after 30, 60, and 150 min were measured [31–33]. To estimate blood glucose level, blood samples of experimental mice were drawn by pricking with a sterile needle in the tail vein. The blood glucose levels were measured by using the glucometre and compatible blood glucose strips. The blood glucose levels were measured in millimole per liter (mmol/L) unit.

### Evaluation of neuropharmacological activity

Open Field and Hole Board test were performed to evaluate neuropharmacological activity of the ethanolic leaves extract of *l. rubiginosa* [34–36].

#### Open field method

In the Open Field method, the test animals were divided into four groups: control, standard and two test samples, consisting of five mice in each group. The control groups received only vehicle (1% tween-80 water) whereas test groups received extract doses of 250 mg/kg and 500 mg/kg body weight of mice. The floor was half square meter open field and it was alternatively colored black and white. The height of wall in the Open Field apparatus was 40 cm. The number of squares traveled by the experimental mice was counted for 3 min. The observations were made on 0, 30, 60, 90 and 120 min after oral administration of control, standard and two test groups. Diazepam (1 mg/kg) was used as a standard drug.

### Hole board method

In the Hole Board method, the test animals were divided into four groups: control, standard and two test samples, consisting of five mice in each group. The control groups received only vehicle (1% tween-80 water) whereas test groups received extract doses of 250 mg/kg and 500 mg/kg body weight of mice. The apparatus consists of an enclosed space having sixteen holes in a grid-pattern. The experimental mice when placed in this apparatus were free to dip its head through the holes. The number of head dipping by the animal was counted for 3 min. The observations were made on 0, 30, 60, 90 and 120 min after oral administration of control, standard and two test groups. Diazepam (1 mg/kg) was used as a standard drug.

### Evaluation of antidiarrheal activity

#### Castor oil induced diarrhea

In Castor oil induced antidiarrheal activity test, the experimental animals were divided into four groups: control, standard and two test samples consisting of five mice of each group. The control groups received only vehicle (1% tween-80 water) whereas test groups received extract doses of 250 mg/kg and 500 mg/kg body weight of mice. Loperamide (3 mg/kg) was used as a standard drug. After 60 min, each mouse of all groups was administered 0.5 ml of castor oil in oral route. All animals were then kept separately in transparent cage having white blotting paper in order to count the no. of faeces. Every hour the blotting paper was changed and it was observed within a period of 4 h. Latent period of faecal drops for each group was also recorded. Latent period of faecal drops and percent inhibition of defecation of each group were determined [37, 38]. By using the following formula percent inhibition of defecation was calculated:

*Percent inhibition* = (*D*_0_ − *D*_1_/*D*_0_) × 100 % 

where D_0_ is the number of defecation of the control group, and D_1_ is the number of defecation of the test or standard group.

### Statistical analysis

All experimental data was expressed as mean ± standard error of mean. Dunnett’s test was used to assess statistical significance by one-way analysis of variance. Statistical analysis was executed in Prism 6.0 (Graph Pad Software Inc., San Diego, CA, USA). Results of the present study were considered as statistically significant when *P* < 0.05.

## Results

### Phytochemical screening

Phytochemical studies showed that carbohydrate, alkaloid, glycoside, phenolic compounds, flavonoids, tannins, steroids and proteins were present whereas combined reducing sugar and gums were absent in the ethanol extract of *L. rubiginosa* (Table 1).

**Table 1:** Result of phytochemical screening

Chemical groups	Extract
Reducing sugar	**+**
Combined reducing sugar	**–**
Tannins	**+**
Flavonoids	**+**
Saponin	**+**
Gums	**–**
Steroids	**+**
Alkaloids	**+**
Glycoside	**+**
Proteins	**+**
Phenolic compounds	**+**

### DPPH-scavenging assay

**Fig. 1:**
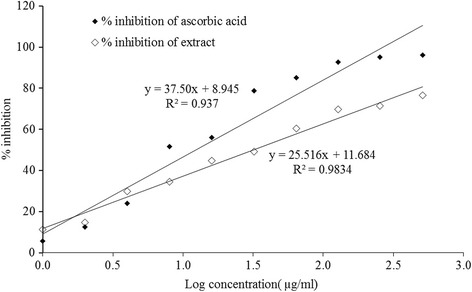
Percent inhibition vs. log concentration graph for standard (ascorbic acid) and extract of *Lepisanthes rubiginosa* L

This assay was carried out to estimate the free radical scavenging activity of the extract. IC_50_ value of *L. rubiginosa* (extract) was found to be 31.62 μg/ml, whereas IC_50_ value of ascorbic acid (standard) was found to be 12.02 μg/ml (Fig. 1).

### Determination of total phenolic content

**Fig. 2:**
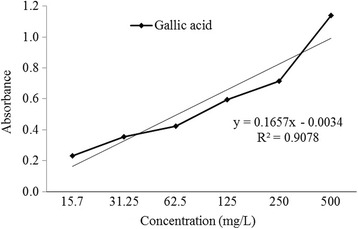
Gallic acid standard calibration curve for the determination of total phenolic content

As shown in Fig. 2, the total phenolic content in *L. rubiginosa* was estimated to be 422.42 mg GAE/100 g of the dry weight extract.

### Determination of total flavonoid content

**Fig. 3:**
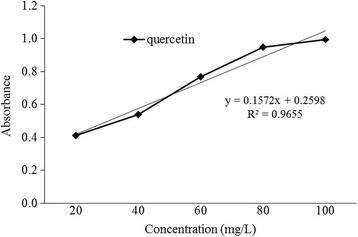
Quercetin standard calibration curve for the determination of total flavonoid content

Total flavonoid content in *L. rubiginosa* was estimated to be 350 mg QE/100 g of the dry weight extract (Fig. 3).

### Determination of total tannin content

**Fig. 4:**
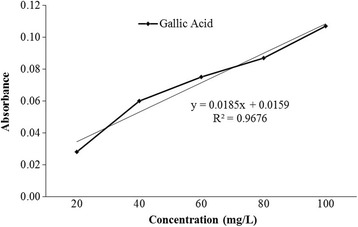
Gallic acid standard calibration curve for the determination of total tannin content

Total tannin content in *L. rubiginosa* was estimated to be 233.3 mg GAE/100 g of the dry weight extract (Fig. 4).

### Evaluation of analgesic activity

**Fig. 5:**
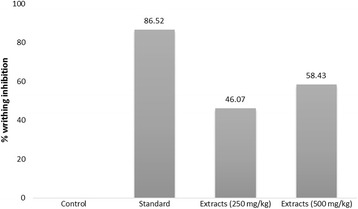
Percentage writhing inhibition of acetic acid induced writhing in mice by the standard drug and *Lepisanthes rubiginosa* L

The results of the test showed that ethanolic leaf extract of *L. rubiginosa* at the dose of 250 mg/kg and 500 mg/kg exhibit significant (*p* < 0.05) inhibition of writhing reflex of 46.07% and 58.43% respectively while the standard (diclofenac-Na 25 mg/kg) drug inhibition was found to be 86.52% (Fig. 5).

### Evaluation of antihyperglycemic activity

The plant extract of *L. rubiginosa* showed significant (*p* < 0.05) antihyperglycemic activity in mice as compared to control groups (Table 2). The extracts showed a noticeable decrease in locomotion by decreasing number of square crossed in the test animals from the 2nd, 3rd, 4th, 5th observation period at both dose levels (250 and 500 mg/kg body weight). The present study elevated the effect of ethanolic extract of *Lepisanthes rubiginosa* Leenh on experimentally induced anti anxiety and depression.

**Table 2:** Effect of the *Lepisanthes rubiginosa* L. ethanolic extract on oral glucose tolerance test (antihyperglycemic activity test) in normal control mice

Group	*n*	Blood glucose level (mean ± SEM)			
		Fasting	30 min	90 min	150 min
Control (1% tween-80 water)	5	7.16 ± 0.75	19.3 ± 0.64	12.76 ± 0.91	9.62 ± 0.53
Standard	5	7.04 ± 0.42	16.44 ± 0.76*	4.24 ± 0.39***	4.20 ± 0.47***
(Glibenclamide, 5 mg/kg)					
Extract 250 mg/kg	5	8.08 ± 0.44	17.68 ± 0.72	5.40 ± 0.68***	5.22 ± 0.61***
Extract 500 mg/kg	5	7.74 ± 0.47	19.20 ± 0.29	4.94 ± 0.29***	4.70 ± 0.46***

Here, *p < 0.05 and ***p < 0.001 when compared with control group. SEM: standard error of mean

### Evaluation of neuropharmacological activity

The present study evaluated the neuropharmacological activity of ethanol extract of *L. rubiginosa* on mice using Open Field method and Hole Board method. The results are shown in Tables 3 & 4. The mice group treated with extracts (test group) showed a significant decrease in number of squares crossed (Table 3) as well as head dipping (Table 4) at both dose levels (250 and 500 mg/kg body weight). The results demonstrated that the extract significantly decreased locomotor activity at the doses used in the experiment. Here we did not find any statistically significant difference between 250 mg/kg and 500 mg/kg with respect to neuropharmacological activity.

**Table 3:** Neuropharmacological effect of *Lepisanthes rubiginosa* L. on mice in Open Field method

Groups	No. of square crossed (mean ± SEM)				
	O min	30 min	60 min	90 min	120 min
Control (1% tween-80 water)	38.5 ± 3.0	35.8 ± 1.9	37 ± 3.8	38.1 ± 2.1	42.2 ± 3.03
Standard (Diazepam 1 mg/kg)	36.3 ± 1.9	25.8 ± 1.8**	22.3 ± 1.9*	18.5 ± 1.6***	16.7 ± 2.1***
Extract 250 mg/kg	42.0 ± 6.8	28.3 ± 1.3*	24.8 ± 2.3*	23.3 ± 2.3**	20.8 ± 1.6***
Extract 500 mg/kg	36.0 ± 2.7	26.5 ± 2.5*	23.0 ± 2.3*	21.0 ± 2.6**	19.9 ± 1.7****

Here, *p < 0.05, **p < 0.01 and ***p < 0.001 when compared with control group. SEM: standard error of mean, *n* = 5

**Table 4:** Neuropharmacological effect of *Lepisanthes rubiginosa* L. on mice in Hole Board method

Groups	No. of head dipping (mean ± SEM)				
	O min	30 min	60 min	90 min	120 min
Control (1% tween-80 water)	26.5 ± 1.04	29.3 ± 2.99	30.8 ± 4.30	31.0 ± 1.47	35.3 ± 2.20
Standard (Diazepam 1 mg/kg)	29.5 ± 2.40	17.8 ± 1.50*	15.3 ± 1.00*	13.3 ± 0.85***	11.0 ± 1.08***
Extract 250 mg/kg	28.8 ± 1.44	23.5 ± 0.65	19.5 ± 1.00*	16.5 ± 1.50**	15.0 ± 1.80***
Extract 500 mg/kg	26.3 ± 1.40	22.5 ± 1.44	18.5 ± 1.50*	15.5 ± 0.96***	10.5 ± 1.20***

Here, *p < 0.05, **p < 0.01 and ***p < 0.001 when compared with control group. SEM: standard error of mean, *n* = 5

### Evaluation of antidiarrheal activity

In the present study the antidiarrheal activity of the test extract was evaluated in castor oil induced diarrheal mice. In the castor oil induced diarrheal mice, the ethanol leaves extract of *L. rubiginosa* at the doses of 250 and 500 mg/kg body weight of mice significantly decreased the total number of faeces as well as delayed the onset of diarrhea in a dose dependent manner when compared to vehicle-treated negative control group. In this experiment Loperamide was used as positive control for antidiarrheal activity. We compared the antidiarrheal effect of the test extract at 500 mg/kg body weight with the effect of 250 mg/kg body weight dose. We found that the antidiarrheal effect at dose 500 mg/kg was significantly higher than that with 250 mg/kg dose and the difference is statistically significant. This finding further suggests that the test extract exerts antidiarrheal activity in a dose dependent manner. Percent inhibition of defecation at doses 250 and 500 mg/kg body weight was 57.89 and 77.19, respectively whereas standard loperamide (3 mg/kg) was 88.59 (Table 5).

**Table 5:** Effects of *Lepisanthes rubiginosa* L. on castor oil-induced diarrhea in mice

Group	*n*	Onset of diarrhea (mean ± SEM, min)	Number of stools after 4 h (mean ± SEM)	Inhibition of defecation (%)
Control (1% tween-80 water)	5	33.0 ± 3.59	22.80 ± 1.63	
Standard (Loperamide 3 mg/kg)	5	197.4 ± 2.65***	2.60 ± 0.50***	88.59
Extract 250 mg/kg	5	101.0 ± 3.64***	9.60 ± 0.74***	57.89
Extract 500 mg/kg	5	178.8 ± 5.00***,†††	5.20 ± 0.58***,††	77.19

Here, *p < 0.05, **p < 0.01 and ***p < 0.001 when compared with control group. ^††^ *p* < 0.01 and ^†††^ *p* < 0.001 when compared with Extract 250 mg/kg group. SEM: standard error of mean

## Discussion

One of the most important pharmacological properties of plants is antioxidant activity. This assay is widely used as a preliminary test which provides information on the reactivity of test compound with a stable free radical. Reactive oxygen species (ROS) or active oxygen species are the activated oxygen of several forms, like hydroxyl ions, hydrogen peroxide and superoxide ions etc. [39]. These ROS play a dynamic healing role in some perilous disorders such as cancers, inflammations, coronary heart diseases, aging, cataracts, neurodegenerative disorders, atherosclerosis [40]. These antioxidant molecules scavenge the ROS and protect cells by antioxidant defense mechanisms [41]. Among several antioxidant potency investigations, DPPH scavenging assay is one of the straight way for free radical scavenging assay. In doing that, DPPH is converted to unpaired (purple color) to paired electron (yellow colored) by reduction to α, α-diphenyl-β-picryl hydrazine. The degree of color change is proportional to the concentration and potency of the antioxidants molecules. It is found that a huge decrease in the absorbance indicates the significant free radical scavenging activity of the compound [42, 43]. In the present study, the extract of *L. rubiginosa* showed a good IC_50_ value which is compared to standard ascorbic acid (Fig. 1) indicating the presence of antioxidant components in the plant extract. The results of phytochemical screening carried out in this study also suggested that the plant contains phytochemical constituents which are capable of donating hydrogen to scavange the free radicals that might cause potential damage.

Phenolic compounds are considered to be a product of secondary metabolites which derived from phenylalanine and tyrosine that occurs in plant. Plant materials with high phenolic contents are being increasingly used in food industry because of their protective activity against oxidative degradation of lipids. Thus they improve food quality and add nutritional value. Phenolic components containing plants also possess hydroxyl groups which have a good scavenging ability [44, 45]. Some previous studies showed the efficiency of phenolics for scavenging radicals that is influenced by their nature of OH group’s substitution, molecular weight and presence of aromatic rings [46]. These properties may explain the possible mechanisms of antioxidant activites of the plant extract. The result (422.42 mg GAE/100 g of dried extract) proved that the extract of *L. rubiginisa* leaves have a handsome amount of phenolic compound which indicates the presence of hydroxyl groups.

Another important category of phenolic compounds are flavonoids which have a good antioxidant activity. Flavonoids are natural compounds occurring in plant and having a positive effect on human health. Flavonoid and its derivatives show a varied range of anti-inflammatory antibacterial, anticancer, antiviral and anti-allergic activities. It is also considered to be highly effective against oxidizing molecules like singlet oxygen and various free radicals responsible for several diseases [47, 48]. In another study, it is shown that flavonoids exhibit their activity by affecting membrane permeability by inhibiting membrane bound enzymes like ATPase and phospholipase A2. These properties may elucidate the conceivable mechanisms of antioxidative action of the extract. Our results indicate that phytochemical constituents present in the extracts contain a good amount of flavonoid compounds (350 mg QE/100 g of the dry extract) which support the usefulness of this plant in folklore remedies in the treatment of various disorders related to stress [49–52].

Tannins are astringent and bitter plant polyphenols. They bind to protein molecules and precipitate or shrink them. Tannins are large polyphenolic compounds containing sufficient hydroxyl groups and other free radicals such as carboxyl and they form complexes with proteins and other macromolecules. These free radicals are very much useful in protecting cell from damage [53]. Some studies indicate that tannins are useful indealing of ulcerated or inflamed tissues and they also have a good activity against cancer preventions [54, 55]. The result suggests that the extract of *L. rubiginisa* contains good amount of tannins which may be a source of free radicals responsible for protecting cell from death and satisfy the probable mechanisms how the plant extract exerts its scavenging activities.

In pathological conditions, tissue injury causes pain resulting in the local release of chemical mediators such as prostaglandins, cytokines, leukotrienes etc. They act on the nerve terminals in both activating them directly and enhancing their sensitivity to other stimulation [56, 57]. Acetic acid administration in intraperitoneal routes can produce pain by consequent abdominal writhing due to the release of mediators like prostaglandin E2 and other lipooxygenase products [58]. Prostaglandin mainly prostacyclines (PGI2) and prostaglandin E (PG-E) are responsible for pain sensation due to the excitation of Að-nerve fibers [50]. Thus, the plant extract of *L. rubiginisa* may produce non-narcotic analgesic activity due to the inhibition of prostaglandin synthesis by blocking of lipooxygenase and cyclooxygenase activities. In the present study, ethanolic leaves extract of *L. rubiginisa* showed a significant (*p* < 0.05) writhing inhibitions compared to that of standard diclofenac-Na. The result also shown that percentage of writhing inhibition in two test samples (250 mg/kg & 500 mg/kg) are sufficient enough to produce analgesic activity as compared to standard test results. As we know that the standard drug diclofenac sodium is a potent analgesic and it can produce stronger analgesic activity rather the ethanolic leaves extract of *L. rubiginosa*.

Diabetes is a metabolic disorder that indicates elevated blood glucose concentration and occurred by an insufficient of insulin secretion and action [59, 60]. The agents that are used to treat diabetics by means of decreasing blood glucose concentration or sufficiently secreting insulin are known as antihyperglycemic agents. In a recent report Joy and his group the mechanism of antihyperglycemic agents had either by potentiating pancreatic insulin secretion or increasing glucose uptake [61]. Such mechanism has also been proposed in another study, root extracts of *Helicteresisora* [62]. The current study of ethanolic leaves extract of *L. rubiginosa* expressed a significant (*p* < 0.05) antihyperglycemic activity as compared to control and other groups (Table 2). The possible mechanism of this activity may be either by potentiating pancreatic insulin secretion or increasing glucose uptake. The result also demonstrated that animal treating with standard drug (Glibenclamide 5 mg/kg) had been shown strong antihyperglycemic activity rather than the other groups and the two test groups (250 mg/kg & 500 mg/kg) also shown a significant decrease in blood glucose level. From the above discussion, we can say that the ethanolic extract of *L. rubiginosa* may be a potential source of antihyperglycemic agents.

The present work has evaluated the neuropharmacological activity of *L. rubiginisa* in two different experimental model, Open Field and Hole Board method. The open field method is a classical method for evaluating anxiety, exploratory behavior and general motor activity. Locomotion means increase in alertness and decrease in locomotor activity is considered as sedative effects [63]. Mice have a normal tendency to spend more time in the protective corners which suggest that the walls confer anxiety-relieving body contact. When animals are placed in Open Field apparatus they express anxiety by decreasing number of square crosses in the open space as well as to spend more time in protective corners due to fear generation. The reason behind the fear generation is to reflect an aversion towards open spaces [64]. Similar observations were shown in our current study. Our extracts at both doses (250 mg/kg & 500 mg/kg) caused a significant suppression of movements resulting in decreased number of square crossed as compared to control groups. Standard drug (diazepam 1 mg/kg) also exerted significant suppression of movements. The results indicated that the extract of *L. rubiginosa* has CNS depression activity.

Studies have shown that the head dipping behavior of mice is directly related to their emotional state. Base on the concept the anxiolytic state of mice may be the reflection of head dipping behavior [65]. Treatment with ethanolic extract at both doses (250 and 500 mg/kg) caused a statistically significant reduction in the number of head dipping when compared to control groups. The standard drug (diazepam 1 mg/kg) also showed noticeable decrease in head dipping. The result of this study proved that the ethanolic extract of *L. rubiginosa* possesses CNS depression activity. In case of anxiety, the number of head dipping in mice is increased but in depression the number of head dipping in mice is decreased. GABA (Gamma-amino-butyric acid) is one of the major inhibitory neurotransmitters in the CNS (central nervous system). CNS depressant drugs show their action by inferring with GABA receptors [66].

Medicinal plants have been investigated for their activity in gut motility, intestinal transit, and water absorption or reduce intraluminal fluid accumulation [67]. Diarrhea refers to excess passage of watery stools which is caused by decreased consistency or increased frequency of bowel movements. The possible mechanism of diarrhea would be the change in active ion transport system either by increasing chloride secretion or decreasing sodium absorption. It may also include the increase in luminal osmolality, change in intestinal motility and increase in tissue hydrostatic pressure [68]. Among the all mechanisms, castor oil (active compound ricinoleic acid) induces diarrhea by stimulating intestinal motility and secretory processes [69]. In the present study, antidiarrheal activity of the plant extract of *L. rubiginisa* was investigated by castor oil-induced diarrhea in mice. In the castor oil induced diarrheal mice, the ethanolic leaf extract of *L. rubiginosa* at the doses of 250 and 500 mg/kg body weight of mice significantly decreased the total number of feces as well as delayed the onset of diarrhea in a dose dependent manner when compared to vehicle-treated negative control group. Loperamide, an antidiarrheal drug, was used as positive control. We also compared the antidiarrheal effect of the two test doses (500 mg/kg vs. 250 mg/kg body weight). We found that the antidiarrheal effect at dose 500 mg/kg was significantly higher than that with 250 mg/kg dose (Table 5). Percent inhibition of defecation at doses 250 and 500 mg/kg body weight was 57.89 and 77.19, respectively whereas with loperamide (3 mg/kg) it was 88.59. This finding further suggests that the test extract exerts antidiarrheal activity in a dose dependent manner. Some other plant extracts have shown the similar effect [70].

## Conclusion

Our results demonstrated the presence of antioxidant, analgesic, antihyperglycemic, neuropharmacological and antidiarrheal activity. The results of these investigations justify the uses of the plant in folk medicine. These preliminary studies do not describe the actual mechanism of which these pharmacological activities were shown. Further advanced investigations are required to identify the actual mechanism as well as to isolate bioactive compounds responsible for each pharmacological activity.

## Additional file

Additional file 1: The ARRIVE Guidelines Checklist. (DOCX 660 kb)
