# Supplementary material for: Phytochemical and pharmacological evaluation of ethanolic extract of Lepisanthes rubiginosa L. leaves
Source: BMC Complement Altern Med. 2017 Nov 22;17:496. doi: 10.1186/s12906-017-2010-y (PMC5700657; doi:10.1186/s12906-017-2010-y)

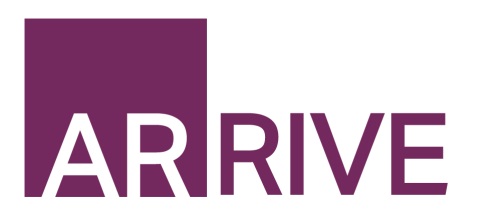


The ARRIVE Guidelines Checklist

Animal Research: Reporting In Vivo Experiments

Carol Kilkenny^1^, William J Browne^2^, Innes C Cuthill^3^, Michael Emerson^4^ and Douglas G Altman^5^

*^1^The National Centre for the Replacement, Refinement and Reduction of Animals in Research, London, UK, ^2^School of Veterinary Science, University of Bristol, Bristol, UK, ^3^School of Biological Sciences, University of Bristol, Bristol, UK, ^4^National Heart and Lung Institute, Imperial College London, UK, ^5^Centre for Statistics in Medicine, University of Oxford, Oxford, UK.*

|  | | ITEM | RECOMMENDATION | Section/ Paragraph |
| --- | --- | --- | --- | --- |
| 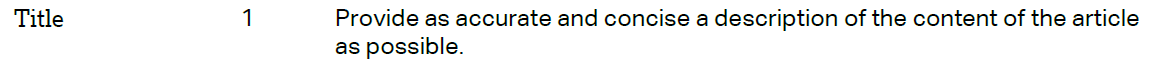 | | | Title |  |
| 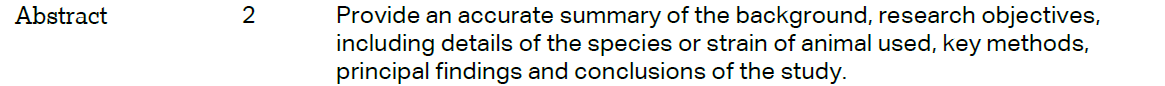 | | | Abstract |  |
| INTRODUCTION | | |  |  |
| 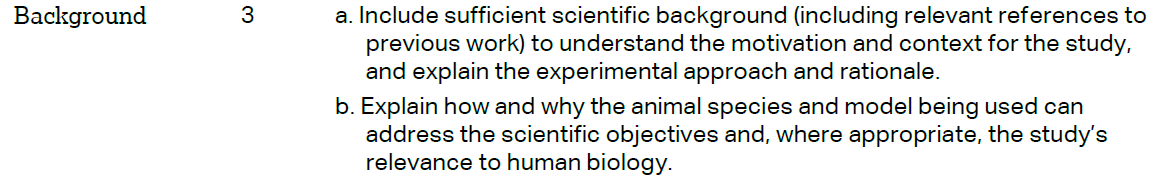 | | | Paragraphs 1-3  Paragraph 2 |  |
| 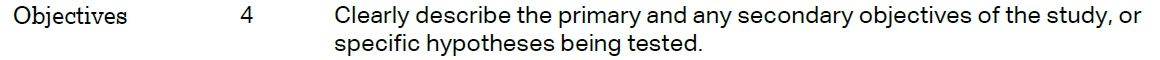 | | | Paragraph 3 |  |
| METHODS | | |  |  |
| 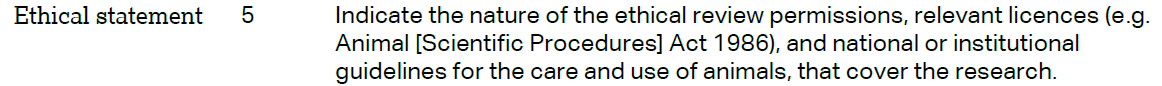 | | | Paragraph 4 |  |
| 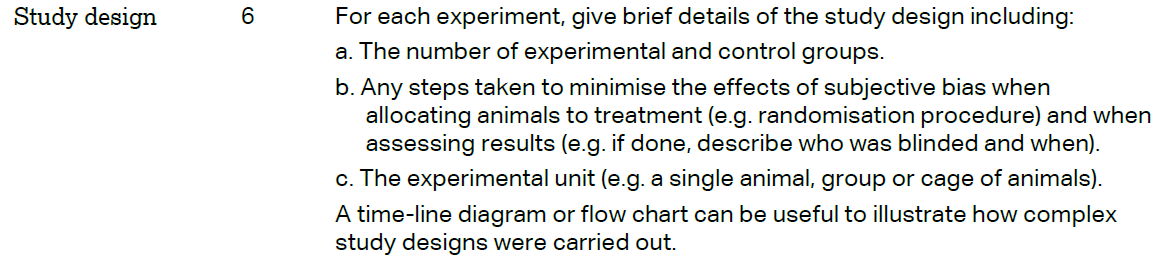 | | | Paragraph 4  Paragraphs 13-17  13-17  13-17 |  |
| 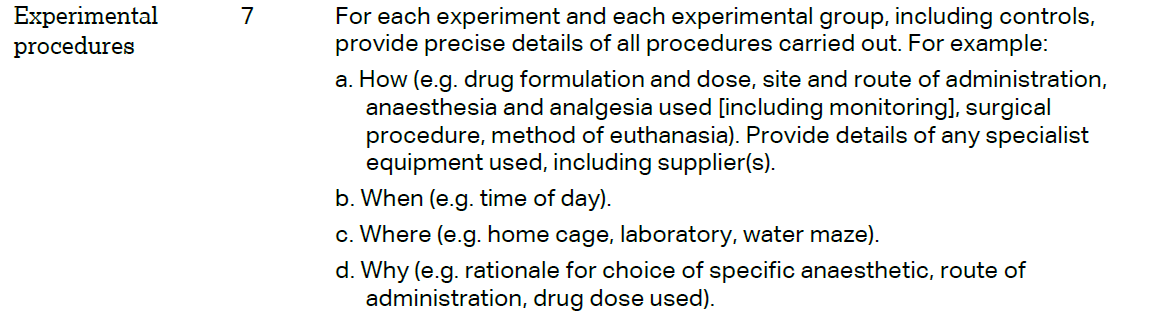 | | | Paragraphs 13-17  13-17  13-17  13-17 |  |
| 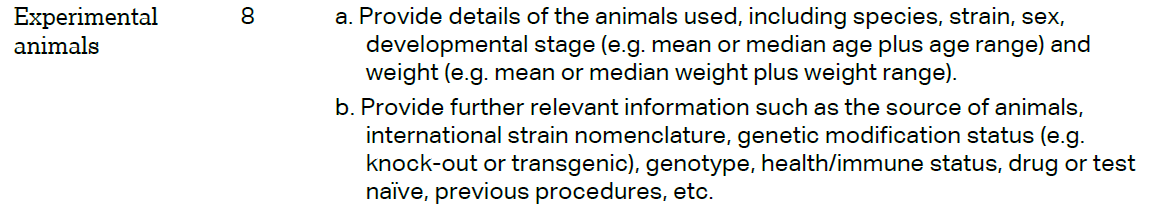 | | | Paragraph 4  Paragraph 4 |  |

The ARRIVE guidelines. Originally published in *PLoS Biology*, June 2010^1^

| 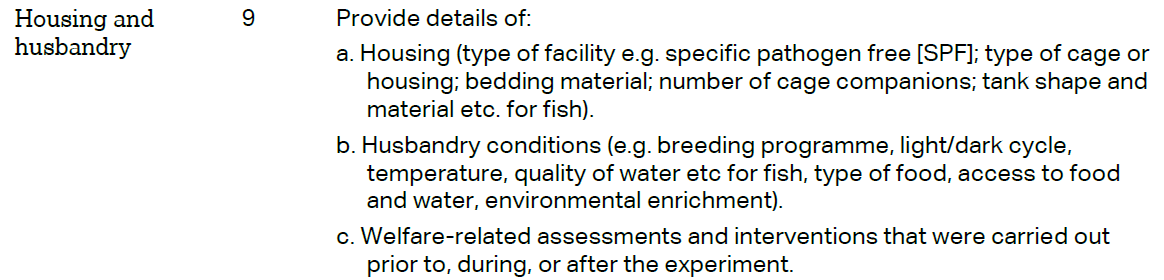 | Paragraph 4 |  |
| --- | --- | --- |
| 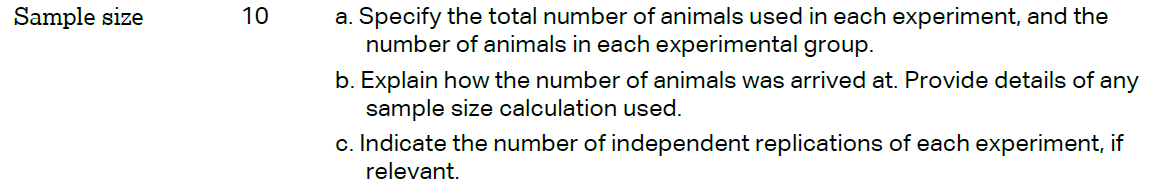 | Paragraph 4  Paragraph 4 |  |
| 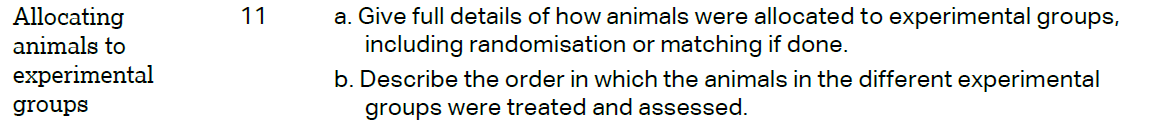 | Paragraphs 13-17  13-17 |  |
| 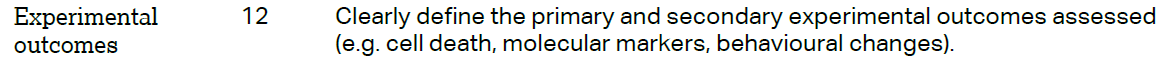 |  |  |
| 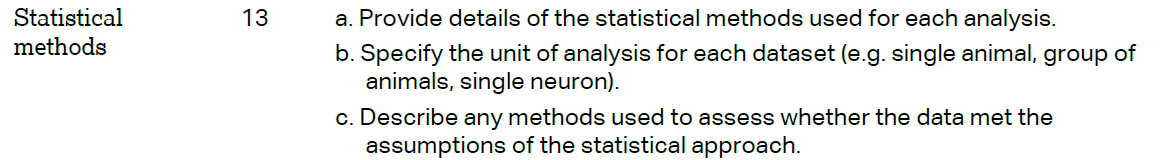 | Paragraph 18  18  18 |  |
| RESULTS |  |  |
| 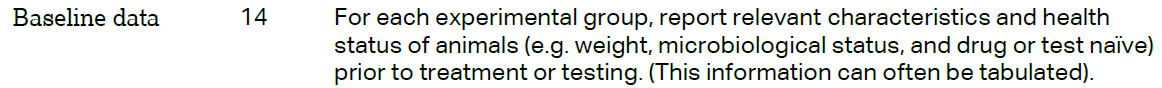 | Paragraphs 13-17 |  |
| 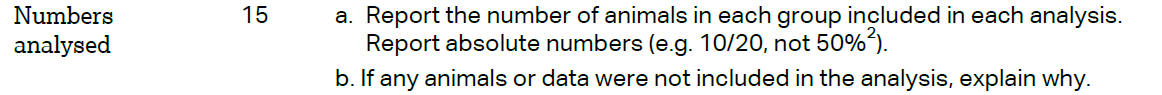 | Paragraphs 13-17 |  |
| 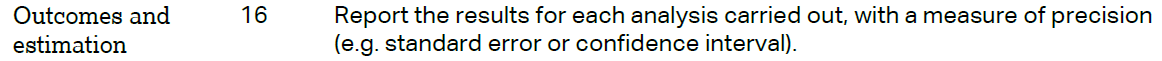 | Paragraphs 6-9 |  |
| 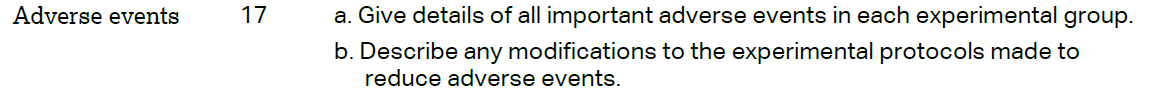 |  |  |
| DISCUSSION |  |  |
| 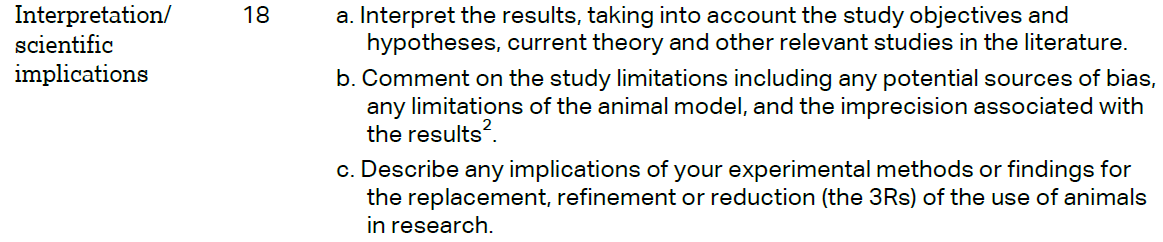 | Paragraphs 1-5  Paragraphs 1-5 |  |
| 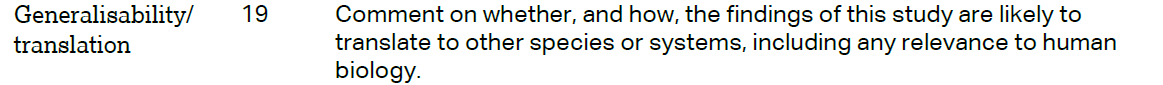 |  |  |
| 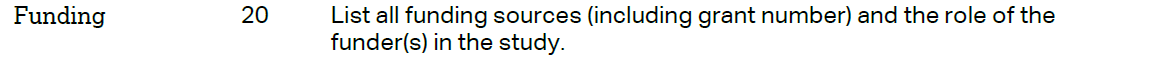 | | Paragraph 7 |


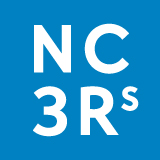

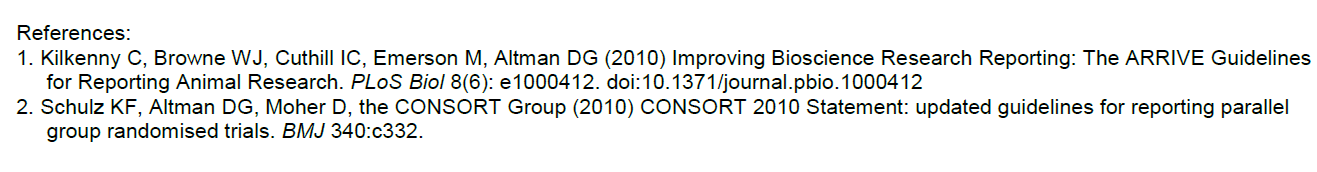

Supplement: Additional file 1: — The ARRIVE Guidelines Checklist. (DOCX 660 kb) [file 12906_2017_2010_MOESM1_ESM.docx]
